# Metagenomic next-generation sequencing for rapid detection of pulmonary infection in patients with acquired immunodeficiency syndrome

**DOI:** 10.1186/s12941-023-00608-9

**Published:** 2023-07-10

**Authors:** Juan Zhong, Yanfen Liu, Na Luo, Qiu Wei, Qisi Su, Jun Zou, Xiaozhong Wu, Xianzhen Huang, Yuting Jiang, Lijuan Liang, Hongmian Li, Jianyan Lin

**Affiliations:** 1 Department of Traditional Chinese Medicine, The First People’s Hospital of Nanning, Nanning, China; 2 The Fourth People’s Hospital of Nanning, Nanning, China; 3 NanNing Center for Disease Control and Prevention, Nanning, China; 4 Nanning Yunju Biotechnology Co., Ltd, Nanning, China; 5 The People’s Hospital of Guangxi Zhuang Autonomous Region, Nanning, China; 6 The First People’s Hospital of Nanning, Nanning, China

**Keywords:** Acquired immunodeficiency syndrome, Pulmonary infection, Metagenomic next-generation sequencing, Diagnosis

## Abstract

**Background:**

Acquired immunodeficiency syndrome (AIDS) is associated with a high rate of pulmonary infections (bacteria, fungi, and viruses). To overcome the low sensitivity and long turnaround time of traditional laboratory-based diagnostic strategies, we adopted metagenomic next-generation sequencing (mNGS) technology to identify and classify pathogens.

**Results:**

This study enrolled 75 patients with AIDS and suspected pulmonary infections who were admitted to Nanning Fourth People’s Hospital. Specimens were collected for traditional microbiological testing and mNGS-based diagnosis. The diagnostic yields of the two methods were compared to evaluate the diagnostic value (detection rate and turn around time) of mNGS for infections with unknown causative agent. Accordingly, 22 cases (29.3%) had a positive culture and 70 (93.3%) had positive valve mNGS results (*P* value < 0.0001, Chi-square test). Meanwhile, 15 patients with AIDS showed concordant results between the culture and mNGS, whereas only one 1 patient showed concordant results between Giemsa-stained smear screening and mNGS. In addition, mNGS identified multiple microbial infections (at least three pathogens) in almost 60.0% of patients with AIDS. More importantly, mNGS was able to detect a large variety of pathogens from patient tissue displaying potential infection and colonization, while culture results remained negative. There were 18 members of pathogens which were consistently detected in patients with and without AIDS.

**Conclusions:**

In conclusion, mNGS analysis provides fast and precise pathogen detection and identification, contributing substantially to the accurate diagnosis, real-time monitoring, and treatment appropriateness of pulmonary infection in patients with AIDS.

## Background

Human immunodeficiency virus (HIV) infection causes acquired immunodeficiency syndrome (AIDS), which is a serious threat to public health. This pathophysiological state reduces and destroys cell-mediated immunity in humans, resulting in a wide range of opportunistic infections by viruses, bacteria, fungi, and parasites. Moreover, AIDS leads to the emergence of complex ecosystems of multiple pathogens, which makes identifying pathogenic mechanisms difficult. For example, of all body’s organs, the lungs are most frequently affected by AIDS-related microbial infections [1, 2]; therefore, respiratory system failure is one of the leading causes of morbidity in patients with AIDS worldwide [3]. Thus, pathologists should proactively consider AIDS-related microbial infections via rapid and accurate microbiological diagnoses to identify more pathogenic microorganisms, explore pathogenic mechanisms, and enable the optimal management and treatment of patients with AIDS.

The traditional growth and isolation of microorganisms from various cultures are limited by the time required, low detection sensitivity, and difficulty of growing pathogens such as *Helicobacter canis* [4], *Helicobacter pylori* [5], *Neisseria meningitidis* [6], and *Bartonella spp.* [7]. In addition, invasive procedures are required when a pathogenic infection is confined to an anatomical location. There has been a rapid development of nucleic acid-based polymerase chain reaction (PCR) technologies, including real-time quantitative PCR and digital PCR, which can detect targeted pathogenic microorganisms at lower concentrations in less time and demonstrate a high degree of sensitivity and precision. However, PCR relies on highly specific primers and fails to detect untargeted genes, which is not conducive for precise treatment. In contrast, metagenomic next-generation sequencing (mNGS, Ion Torrent PGM, Roche’s 454 pyrosequencing technology, Illumina Miseq and Illumina Hiseq) has the potential to overcome the many limitations of conventional diagnostic approaches, and it has many advantages. Theoretically, mNGS-based diagnostic strategies allow for the detection, classification, serology, and antimicrobial resistance characterization of all classes of infectious agents [8]. Technically, mNGS is more precise, accurate, and sensitive, as has been shown and validated in a few clinical studies [9, 10]. More importantly, it greatly benefits immunocompromised patients in terms of a fast diagnosis, preventing their condition from worsening, shortening their hospital stay, and even improving survival rates [8]. Over the last two decades, mNGS technology has been extensively applied in research on the pathogenesis of infectious diseases. For example, accumulating evidence demonstrates the role of pathogenic microorganisms in periodontal disease [11], chronic/acute inflammation [12–14], cancer [15], metabolic disorders, and neurological diseases [16]. Furthermore, mNGS-based molecular diagnosis is more suitable when a causative etiological agent is suspected but no pathogen is detected through traditional laboratory-based diagnostic strategies, especially culture-based diagnostic methods.

Few studies have comprehensively evaluated the overall diagnostic performance of mNGS in AIDS-related pulmonary infections. Research on the implementation of mNGS is required to compare it with traditional because it must be compared with culture-based approaches and to determine the feasibility of transforming it into a clinical diagnostic test should be discussed. Here, mNGS diagnosis was used to evaluate its value in the efficient and accurate identification of pathogenic microorganisms in patients with AIDS and pulmonary infections. The results mNGS, including precise classification, positive detection rate, turnaround time, and diagnostic positivity rate, were compared with those from conventional culture-based screening. We also revealed the characteristics of the pathogen spectrum in 75 patients with AIDS and 32 patients without AIDS using mNGS. Further exploration of the value of mNGS in detecting co-infections in patients is necessary. Our results demonstrate that mNGS provides a new opportunity to investigate the pathogens associated with AIDS-associated pulmonary infections.

## Materials and methods

### Patients and culture-based assays

A total of 107 individuals with pulmonary infection, aged 19–97 years, participated in this study. Of these, 75 patients with AIDS were recruited from Nanning Fourth People’s Hospital, and 32 participants without AIDS were recruited from Nanning First People’s Hospital, China. AIDS was diagnosed on the basis of the diagnostic criteria and principles of HIV/AIDS (National Center for AIDS/STD Control and Prevention, China Centers for CDC and Prevention). AIDS with a CD4 + T lymphocyte count < 200 /µL may be accompanied by an unexplained irregular fever at 38 °C lasting for more than 1 month, diarrhea for 1 month (i.e., bowel movement more than three times per day), weight loss of more than 10% within 6 months, and typical bacterial, fungal, and viral infections. Prior to the study, all eligible participants and their legal guardians received a full explanation of the study and provided written informed consent. Bronchoscopy-guided puncture sputum and bronchoalveolar lavage fluid samples with suspected pulmonary infections were collected at Nanning Fourth People’s Hospital between January 2020 and May 2021. The samples were promptly stored in sterile containers, sent to the microbiology laboratory, and placed at -80 °C before performing culture-based assays and mNGS-related analysis.

Further bacterial and fungal culture-based assays were performed according to the standard procedure of microbial identification using culture-based assays v4.0 (JYK -SOP-WSW-014, Nanning Fourth People’s Hospital, Guangxi, China). Bronchoalveolar lavage fluid and sputum samples were added to an appropriate volume of culture broth and incubated at 30 °C for 12 h. Subsequently, 100 µL of each dilution (100 and 1,000 colony forming units [CFU]/mL) of each culture was plated on MacConkey agar (Oxoid Ltd., Basingstoke, United Kingdom), sheep blood agar (Oxoid Ltd., Basingstoke, United Kingdom), and chocolate agar (Difco, BD, Le Pont de Claix, France). After incubation at 35 °C for 16 h, the number of CFUs in each plate was counted and recorded using a Synbiosis Protocol 3 automatic colony counter (Synbiosis, Frederick, MD, USA). Individual colonies were purified thrice using the corresponding selective plates. All preliminary detected individual colonies were identified and classified using gram stain, catalase tests, and oxidase tests (Oxoid Ltd., Basingstoke, United Kingdom) and the VITEK-2 COMPACT system (BioMérieux, Marcy-l’Etoile, France) according to the manufacturer’s instructions. *Mycobacterium spp*. were detected using the BacT/ALERT 3D system (BioMérieux, Marcy-l’Etoile, France) and Xpert MTB (Cepheid, Sunnyvale, CA, USA), according to the manufacturers’ instructions. Bronchoalveolar lavage fluid and sputum samples were added to an appropriate volume of Sabouraud agar (pH 4.0–6.0, BioMérieux, Marcy-l’Etoile, France) and incubated at 28 °C for 7 days. If a typical colony grew, suspected colonies of the pathogens were subcultured on CHROMagar Candida agar (CHROMagar, France). Presumptive identification of clinically relevant *Candida spp*. was performed according to colony color and morphological differences. Individual white colonies were further classified using the ID 32 Fungus test kits (BioMérieux, Marcy-l’Etoile, France). Suspected cases of *Pneumocystis spp*. were diagnosed using Giemsa-stained smears, methylene blue staining, and Gomori methenamine silver staining.

### Preparation of genomic DNA

Prior to DNA extraction, an equal volume of sodium hydroxide (NaOH) (40 mg/mL) was added to the sputum, thoroughly mixed, and incubated at 25 °C for 30 min. The mixture was then centrifuged at 25 °C for 10 min at 13,000 rpm, and the pellet was washed twice with physiological saline solution. In contrast, we mixed 0.5 mL of bronchoalveolar lavage fluid with 1 g of 0.5 mm glass beads (TUOTAINUO, Shenzhen, China) in a 1.5 mL centrifuge tube (KIRGEN, Shanghai, China) that was placed on the horizontal plate of the vortex (Kylin-Bell Vortex-5, Haimen, China) at 2,800–3,200 rpm for 30 min. After that, we separated 0.5 mL of bronchoalveolar lavage fluid into a new 1.5 mL centrifugal tube. DNA was extracted from the pellet and tissue using a TIANamp Micro DNA Kit (TIANGEN BIOTECH, China) according to the manufacturer’s instructions. Finally, the DNA was stored at -20 °C before sequencing.

### Identification of bacterial pathogens using the mNGS approach

Whole mNGS was performed using an Illumina NextSeq 550 DX sequencer (Illumina, San Diego, CA, USA) according to the manufacturer’s protocol. Briefly, fragmentation of the extracted DNA was performed to generate 300 bp fragments using Covaris LE220 (Covaris, Inc., Woburn, MA, USA). Subsequently, the DNA fragments were subjected to end-repair, phosphorylation, A-tailing reactions, and Illumina adapter ligation. Subsequently, DNA libraries were constructed using the Nextera XT Library Construction Kit (Illumina, San Diego, CA, USA) and cleaned using a Magnetic Beads Kit (MAGEN Guangzhou, CHINA). The library size was analyzed using the Agilent 2100 Bioanalyzer system (Agilent Technologies, Santa Clara, CA, USA), and accurate quantification was performed by qualitative PCR using a Bio-Rad CFX96 PCR System (Bio-Rad Laboratories, Hercules, CA, USA). Finally, the qualified libraries were sequenced using Nextseq 550 platform (Illumina, San Diego, CA, USA) for 75 bp single-end reads, and raw sequencing files were uploaded for further analysis.

Upon generation of the sequencing results, FastQC (http://www.bioinformatics.babraham.ac.uk/projects/fastqc/) was used for quality control of the raw data from sequencing by trimming low-quality reads and Illumina adapters. Subsequently, host reads were excluded after alignment of the human genome (Homo sapiens version: GRCh38) using Burrows-Wheeler Alignment (http://bio-bwa.sourceforge.net/) [17]. The remaining non-human sequence data were mapped and classified into the unique pathogenic microorganism database of Guangzhou Sagene Biology for bacterial, viral, fungal, and parasitic species. This database, carrying medical microbiological information, obtains references from the NCBI database (fp://ncbi nlm.nih.gov/genomes), the Ensemble database (http://ensemblgenomes.org/), Virus Resource database (Virus get Variation Resource), JGI Fungi Porta (http://genome.jgi.doe.gov) and other authoritative microorganism databases. A microbial standard was used for verification and optimization. The depth and coverage of each species were calculated using Soap Coverage software (http://soap.genomics.org.cn/). The base number was calculated using the formula “read number * read length,” after which a clinical test report was generated.

### Statistical analyses

Comparisons on clinical characteristics between two groups were conducted according to one-way analysis of variance (ANOVA). A k-mer-based metagenomic taxonomic classifier named centrifuge was used to identify the pathogens in each sample [18]. The mNGS results were considered as microorganisms with pulmonary pathogenicity if one of the following criteria were met [45]. A detected single species of pathogenic bacteria, virus or fungi were considered positive if at least 10 unique reads were aligned to the reference genome. mNGS reported a bacterium, virus and parasite whose unique reads at species level scored at least 10-fold greater than that of any other microbes, and reported a fungi whose unique reads at species level scored at least 5-fold greater than that of any other fungus. Although only one unique read was aligned to the reference genome of *Mycobacterium tuberculosis* using mNGS, the results of clinical nucleic acid tests were positive [6, 19, 20]. *Mycobacterium avium*, *e* and *Mycobacterium abscessus* were defined as positive when the number of unique reads (at the genus or species level) was in the top 10 of the reported bacterial list. Microbes identified using mNGS were classified as confirmed pathogens if they were positive in any clinical test (including culture, smear, or Xpert). Microbes were considered potential pathogens if they were identified as positive infectious pathogens using only mNGS but not any other clinical test. All other microbes identified using mNGS were considered uncertain pathogens, but did not meet the criteria for a positive mNGS result. The detection rates of all the pathogens were verified and reported.

## Results

### Comparison of microbiota detected from culture-based assays and mNGS

Based on the inclusion and exclusion criteria, 75 patients with AIDS and 32 patients without AIDS were recruited. The demographic and clinical characteristics of both the groups are presented in Table 1. There were no significant differences in gender proportion (*P* value = 0.069, Chi-square test). However, the level of lymphocyte, neutrophil, white blood cell, C-reactive protein, and procalcitonin in the group of patients without AIDS were significant higher than those of the AIDS group (*P* value < 0.05, one-way ANOVA).

**Table 1:** Clinical characteristics (age, gender, Lymphocyte, Neutrophil, White blood cell, C-reactive protein and Procalcitonin) of enrolled AIDS patients and non-AIDS participants

Variables	Patients with AIDS (n = 75)	Patients without AIDS (n = 32)	*P* value
Age (years)	51.7 ± 14.3	63.2 ± 21.0	< 0.01
Gender (male vs. female)	59:16	19:13	0.069
Lymphocyte (10^9^/L)	1.00 ± 0.75	1.58 ± 1.92	0.026
Neutrophil (10^9^/L)	3.53 ± 2.55	14.05 ± 13.00	< 0.01
White blood cell (10^9^/L)	5.10 ± 2.69	16.27 ± 11.41	< 0.01
C-reactive protein (mg/L)	29.45 ± 36.5	110.96 ± 69.82	< 0.01
Procalcitonin (ng/mL)	0.22 ± 0.29	7.95 ± 23.96	< 0.01

Notes: Student’s t-test and chi-square test for continuous and discrete variables, respectively. A p value less than 0.05 was considered statistically significant

The bronchoalveolar lavage fluids of all participants were clinically tested using culture and Giemsa-stained smear screening. Table 2 includes the data for the detection of bacteria, fungi, and viruses from the samples. Among them, the positive detection rates of the culture-based diagnostic approach and Giemsa-stained smear screening were obtained from 22 (29.3%) and 6 (8.0%) patients, respectively. According to the bacteriological results, *Mycobacterium spp*. (n = 10, 13.3%), *Haemophilus influenzae* (n = 2, 2.7%), *Escherichia coli* (n = 1, 1.3%), *Klebsiella pneumoniae* (n = 1, 1.3%), *Rhodococcus equi* (n = 1, 1.3%), and gram-negative bacteria (n = 3, 4.0%) were culture-positive on the bacterial growth plates mentioned above. One of them (sample ID: B2102820_AA) was simultaneously infected with both *E. coli* and *Mycobacterium tuberculosis*. In contrast, two (2.7%) cases of *Talaromyces marneffei*, two (2.7%) *Aspergillus spp.*, and one (1.3%) of each of *Candida albicans*, *Candida glabrata*, and *Candida krusei* were detected on CHROMagar Candida agar. One of these samples (sample ID: B2102121_AA) was co-infected with both *C. glabrata* and *C. krusei*. In addition, six cases of *Pneumocystis spp.* were reported to be positive by Giemsa-stained smear screening.

**Table 2:** Distribution of diagnoses based on culture, Xpert and mNGS confirmation of species identity of isolates

Sample Id	Sample type	Microbial classification	mNGS-positive results	Total reads	Culturing-positive results	Xpert
B2106316_AA	Bronchoalveolar lavage fluid	Gram-positive bacteria	*Mycobacterium tuberculosis	600	*Mycobacterium spp.*	*Mycobacterium tuberculosis*
		Fungi	*Pneumocystis jirovecii*	1		
		dsDNA virus	*Human gammaherpesvirus 4*	304		
BH211504_AA	Bronchoalveolar lavage fluid	Fungi	*Pneumocystis jirovecii*	32	*Talaromyces marneffei*	NA
		Fungi	*Penicillium marneffei*	12,298		
		dsDNA virus	*Human cytomegalovirus*	7		
		dsDNA virus	*Human gammaherpesvirus 4*	4948		
BH211505_AA	Bronchoalveolar lavage fluid	Gram-positive bacteria	*Rothia slime*	14,959	*Mycobacterium spp.*	NA
		Gram-negative bacteria	*Isoeveillonella spp.*	11,915		
		Gram-negative bacteria	*Prevotella niger*	55,489		
		dsDNA virus	*Human gammaherpesvirus 4*	4		
B2103830_AA	Bronchoalveolar lavage fluid	Gram-positive bacteria	*Streptococcus parasanguis*	1893	*Haemophilus influenzae*	NA
		Gram-positive bacteria	*Streptococcus pneumoniae*	705		
		Gram-negative bacteria	*Haemophilus parainfluenzae*	1019		
		Gram-negative bacteria	*Haemophilus influenzae	437		
		Fungi	*Candida albicans*	9		
		Fungi	*Pneumocystis jirovecii*	1		
		dsDNA virus	*Human gammaherpesvirus 4*	9		
		dsDNA virus	*Human cytomegalovirus*	4		
B2103362_AA	Bronchoalveolar lavage fluid	Gram-positive bacteria	*Streptococcus pneumoniae*	718	*Candida albicans*	NA
		Gram-positive bacteria	*Oral Streptococcus*	2178		
		Gram-negative bacteria	*Klebsiella pneumoniae*	84		
		Fungi	*Candida albicans	5024		
		dsDNA virus	*Human gammaherpesvirus 4*	6		
B2102995_AA	Bronchoalveolar lavage fluid	Gram-positive bacteria	*Tropheryma whipplei*	13,336	*Talaromyces marneffei*	NA
		Gram-positive bacteria	*Streptococcus pneumoniae*	917		
		Fungi	*Penicillium marneffei*	35		
		dsDNA virus	*Human gammaherpesvirus 4*	55		
B2102820_AA	Bronchoalveolar lavage fluid	Gram-negative bacteria	*Enterobacter cloacae*	849	*Escherichia coli and Mycobacterium spp.*	NA
		Gram-negative bacteria	*Pseudomonas aeruginosa*	1119		
		Gram-negative bacteria	*Escherichia coli	23,469		
		Gram-negative bacteria	*Klebsiella pneumoniae*	134,747		
		dsDNA virus	*Human gammaherpesvirus 4*	11,103		
		dsDNA virus	*Human cytomegalovirus*	160		
B2102821_AA	Bronchoalveolar lavage fluid	Gram-negative bacteria	*Klebsiella pneumoniae	64,903	*Klebsiella pneumoniae*	NA
		Fungi	*Candida albicans*	3		
		dsDNA virus	*Human gammaherpesvirus 4*	98		
B2102822_AA	Bronchoalveolar lavage fluid	Gram-positive bacteria	*Streptococcus pneumoniae*	1451	*Mycobacterium spp.*	NA
		Gram-positive bacteria	*Streptococcus parasanguis*	2830		
		Gram-positive bacteria	*Mycobacterium avium	4		
		dsDNA virus	*Human cytomegalovirus*	16		
B2102519_AA	Bronchoalveolar lavage fluid	Fungi	*Pneumocystis jirovecii*	129	*Aspergillus spp.*	NA
		dsDNA virus	*Human cytomegalovirus*	168		
		dsDNA virus	*Human gammaherpesvirus 4*	21		
B2102520_AA	Bronchoalveolar lavage fluid	Gram-negative bacteria	*Klebsiella pneumoniae*	227	*Gram-negative bacteria*	NA
		Gram-negative bacteria	*Helicobacter pylori*	321		
		Fungi	*Penicillium marneffei*	157		
		dsDNA virus	*Human gammaherpesvirus 4*	48,541		
B2102121_AA	Bronchoalveolar lavage fluid	Gram-positive bacteria	*Staphylococcus aureus*	122	*Gram-negative bacteria, Candida glabrata and Candida krusei*	NA
		dsDNA virus	*Human gammaherpesvirus 4*	6038		
		Gram-positive bacteria	*Streptococcus pneumoniae*	3016		
		Gram-positive bacteria	*Streptococcus parasanguis*	3822		
		Gram-negative bacteria	*Klebsiella oxytoca	3375		
		Fungi	*Candida glabrata	2452		
		Fungi	*Pichia kudri azwei*	231		
		Fungi	*Penicillium marneffei*	144		
		Fungi	*Aspergillus fumigatus*	12		
		dsDNA virus	*Human cytomegalovirus*	8		
B2101782_AA	Bronchoalveolar lavage fluid	Gram-positive bacteria	*Tropheryma whipplei*	11,657	*Haemophilus haemophilus*	NA
		Gram-positive bacteria	*Pseudostreptococcus pneumoniae*	9		
		Gram-positive bacteria	*Streptococcus pneumoniae*	8		
		Gram-negative bacteria	*Haemophilus influenzae*	27,396		
		Fungi	*Aspergillus fumigatus*	15		
B2101349_AA	Bronchoalveolar lavage fluid	Gram-positive bacteria	*Mycobacterium kansasii	39	*Mycobacterium spp.*	NA
		V:DNA	*Human gammaherpesvirus 4*	153		
B2100619_AA	Bronchoalveolar lavage fluid	Fungi	*Pneumocystis jirovecii*	70	*Mycobacterium spp.*	*Mycobacterium tuberculosis*
		V:DNA	*Human gammaherpesvirus 4*	359		
		V:DNA	*Human cytomegalovirus*	10		
B2100621_AA	Bronchoalveolar lavage fluid	Gram-positive bacteria	*Mycobacterium tuberculosis	29	*Mycobacterium spp.*	NA
		Fungi	*Candida albicans*	1316		
		V:DNA	*Human gammaherpesvirus 4*	23		
B2100395_AA	Bronchoalveolar lavage fluid	Gram-positive bacteria	*Enterococcus faecium*	262	*Aspergillus spp.*	NA
		Gram-positive bacteria	*Streptococcus pneumoniae*	3		
		Gram-negative bacteria	*Pseudomonas aeruginosa*	3		
		Fungi	*Aspergillus fumigatus*	3336		
		Fungi	*Aspergillus flavus*	68		
		V:DNA	*Human gammaherpesvirus 4*	490		
		V:DNA	*Human cytomegalovirus*	7		
B2100396_AA	Bronchoalveolar lavage fluid	Gram-positive bacteria	*Streptococcus pneumoniae*	716	*Rhodococcus equi*	NA
		Gram-positive bacteria	*Rhodococcus equi	10,291		
		V:DNA	*Human gammaherpesvirus 4*	21		
B2100107_AA	Bronchoalveolar lavage fluid	Gram-negative bacteria	*Klebsiella pneumoniae	665	*Gram-negative bacteria*	NA
B2006273_AA	Bronchoalveolar lavage fluid	Gram-positive bacteria	*Mycobacterium tuberculosis	18	*Mycobacterium spp.*	NA
		V:DNA	*Human gammaherpesvirus 4*	18		
B2006130_AA	Bronchoalveolar lavage fluid	V:DNA	*Human cytomegalovirus*	4	*Mycobacterium spp.*	NA
B2006023_AA	Bronchoalveolar lavage fluid	Gram-positive bacteria	*Oral Streptococcus*	36	*Mycobacterium spp.*	NA
		Gram-positive bacteria	*Nocardia beijing*	512		
		Gram-positive bacteria	*Streptococcus pneumoniae*	11		
		V:DNA	*Human gammaherpesvirus 4*	143		

Note: Sample Id, microbial classification, mNGS-positive results, the number of unique reads and culturing-positive results were revealed and * means confirmed pathogens with pulmonary pathogenicity when # means potential pathogenic agents

We evaluated the detection performance of the traditional culture-independent screening and mNGS. Notably, 15 patients with AIDS showed concordant results between culture and mNGS, whereas 5 showed discordant results. Concordance between mNGS and culture for the detection of *Mycobacterium spp*. (5 cases) was found, including 3 cases (B2106316_AA, B2100621_AA and B2006273_AA) of *Mycobacterium tuberculosis*, 1 case (B2101349_AA) of *Mycobacterium kansasii* and 1 case (B2102822_AA) of *Mycobacterium avium*. However, discordant results between culture-positive and mNGS-negative samples (sample ID: BH211505_AA, B2100619_AA, B2102820_AA, B2006130_AA, and B2006023_AA) of *Mycobacterium tuberculosis*. were strongly associated with limitations in sequencing coverage. Moreover, consistent results for the identification of *Haemophilus haemophilus* (sample ID: B2103830_AA and B2101782_AA), *E. coli* (sample ID: B2102820_AA), *K. pneumoniae* (sample ID: B2102821_AA), and *R. equi* (sample ID: B2100396_AA) between the two detection methods were also found in our study. Furthermore, the positive results for *T. marneffei* (samples ID: BH211504_AA and B2102995_AA), *Candida spp.* (samples - ID: B2103362_AA and B2102121_AA), and *Aspergillus fumigatus* (sample ID: B2100395_AA) were validated by both culture and mNGS. Regarding the detection of *P. jirovecii*, only one case showed concordant results between Giemsa-stained smear screening and mNGS. The other five Giemsa-stained smears that screened positive remained negative on mNGS. Pathogens (*Mycobacterium tuberculosis, Haemophilus influenzae, e, Escherichia coli, eeMycobacterium avium, Helicobacter pylori, Candida glabrata, Mycobacterium kansasii, Aspergillus fumigatus* and *Rhodococcus equi*) eboth mNGS and clinical tests (including culture, smear, or Xpert) were considered as confirmed microorganisms with pulmonary pathogenicity (Table 2).

**Fig. 1:**
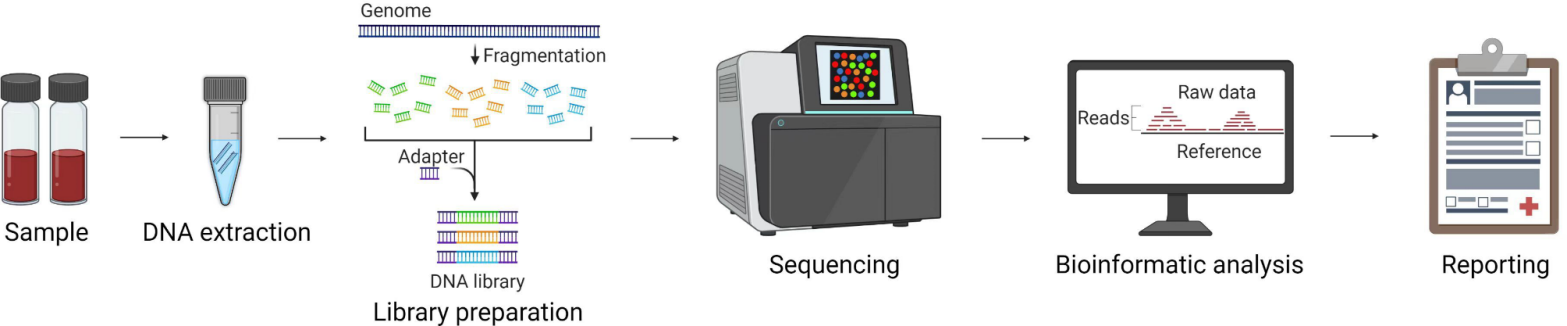
Technical roadmap and flow diagram for pathogenic detection based on mNGS

In our investigation, both gram-negative and gram-positive bacteria took 3–5 days to culture and classify before report generation. Furthermore, identification of *Mycobacterium spp.* and fungi took more than 14 days. In contrast, the total process of mNGS-based detection, including nucleic acid extraction, library construction, high-throughput sequencing, bioinformatics analysis, and reporting, required only 16 h (Fig. 1).

Notably, many other potential pathogens of lung infections in 75 patients with AIDS were identified using mNGS, which provided a wide range of profiles of pathogenic microorganisms. Based on annotations obtained from the pathogenic microorganism database, the percentage of mNGS-positive results was 70/75 (93.3%). There were 47 (62.7%) bacterial infections, 34 (45.3%) fungal infections, and 57 (76.0%) viral infections. The positive detection rates for gram-negative and gram-positive bacteria were 23/75 (30.7%) and 38/75 (50.7%), respectively. In addition, the detection rate of multipathogenic infections (at least three types of pathogenic microorganisms) was 45/75 (60.0%). When analyzed using mNGS, *Human gammaherpesvirus* 4 (n = 39, 52.0%) was the most prevalent pathogen found in patients with AIDS and lung infection, followed by *Streptococcus pneumonia*e (n = 27, 36.0%), *Human cytomegalovirus* (n = 25, 33.3%), *P. jirovecii* (n = 22, 29.3%), *Penicillium marneffei* (n = 12, 16.0%), *H. influenza*e (n = 9, 12.0%), *Streptococcus mitis* (n = 8, 10.7%), *K. pneumoniae* (n = 7, 9.3%), *Tropheryma whipplei* (n = 6, 8.0%), and *Mycobacterium spp*. (n = 5, 6.7%). For AIDS patients, *Human gammaherpesvirus 4*, *Streptococcus pneumoniae*, *Penicillium marneffei* and *Human cytomegalovirus* were typical potential pathogens. The presence of *Streptococcus angina*, *Cryptococcus neoformans, human parvovirus B19*, *human herpesvirus 8*, *Fusobacterium nucleatum, Ureaplasma urealyticum*, *Actinomyces grivenis, and Mycoplasma pneumoniae* detected using mNGS in only a single patient was considered false positives because of the low level of reads and lack of validation.

### Characteristics of pathogens spectrum in patients with AIDS revealed by mNGS

**Fig. 2:**
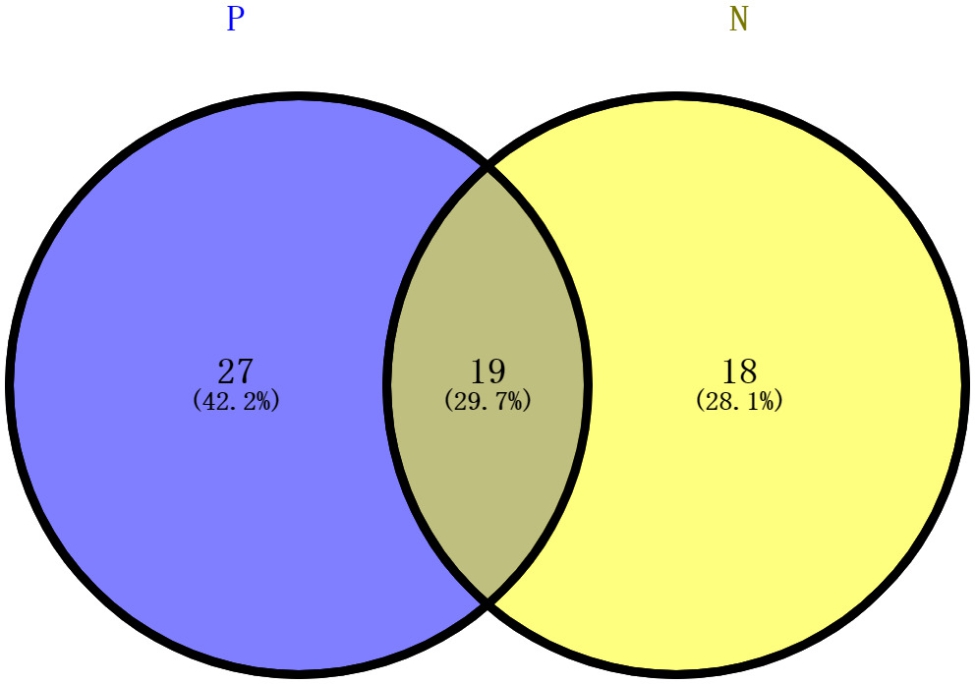
Venn diagram of shared and specific genera. N means without AIDS group. P means AIDS group

**Fig. 3:**
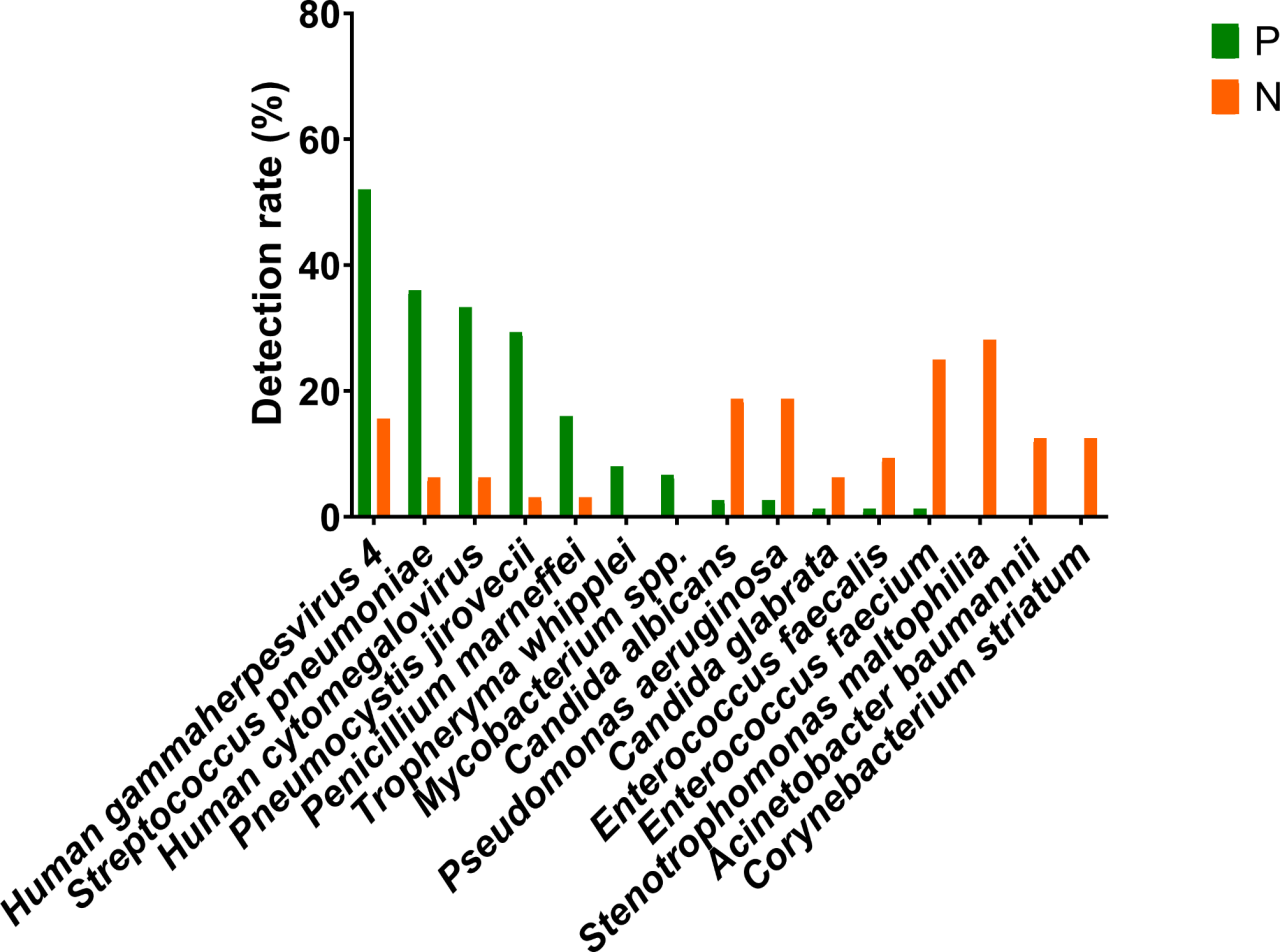
Differences in the profiles of pathogenic microorganisms between 75 patients with AIDS and 32 without AIDS. N means without AIDS group. P means AIDS group. The detection rate of each pathogenic microorganism with pulmonary pathogenicity was calculated

We compared the differences in the profiles of pathogenic microorganisms between 75 patients with AIDS and 32 patients without AIDS. As shown in Fig. 2, we identified 46 types of pathogens in patients with AIDS, whereas 37 were identified in those without AIDS. When further analysis was performed, 18 common pathogen members were consistently detected in both AIDS and non-AIDS groups. We also compared positive detection rates between the patients with AIDS and the 32 without AIDS. As shown in Fig. 3, *Stenotrophomonas maltophilia* (n = 9, 28.1%) was the most prevalent pathogen in patients without AIDS, followed by *Enterococcus faecium* (n = 8, 25.0%), *Pseudomonas aeruginosa* (n = 6, 18.8%), *Helicobacter gammaherpesvirus* 4 (n = 5, 15.6%), *Acinetobacter baumannii* (n = 4, 12.5%), *Corynebacterium striatum* (n = 4, 12.5%), and *Aspergillus fumigatus* (n = 4, 12.5%). When compared with the mNGS-positive results in patients with AIDS, the detection rates of *H. gammaherpesvirus* 4, *S. pneumoniae, Human cytomegalovirus, and P. jirovecii* were approximately decreased 36.4%, 29.8%, 27.1%, and 26.2%, respectively.

## Discussion

Among the microorganisms that cause AIDS-related opportunistic infections, *R. equi* [21–23], *S. pneumoniae* [24–26], *E. coli* [27], *Mycobacterium spp.* [28], *Candida spp.* [29, 30], *P. marneffei* [31, 32], *Aspergillus spp.* [33], *H. gammaherpesvirus 4* [34], *hepatitis B* [35], and *hepatitis C* [35] are frequently identified. When AIDS-related pulmonary infection occurs, the delay in diagnosis may impede precise therapy and block management, leading to poor prognosis and increased mortality and morbidity. Thus, infectious diseases should be accurately diagnosed to guide proper follow-up therapy and prevent cross-infections. Currently, the most common microbial diagnoses for pathogens in specimens are through culture (a gold standard in microbiology for the analysis of microbiota), histopathology, and smear microscopy [36, 37]. In this study, we found that only 22 patients with AIDS were infected with at least one species via culture-based identification, such as *Mycobacterium spp*., *H. influenza*, *E. coli*, *K. pneumoniae*, and *R. equi*. We also validated that the spectrum of pulmonary infection-related pathogens in patients with AIDS was wider than that in patients without AIDS (Fig. 2). Generally, there is a progressive decline in CD4^+^ levels in AIDS, which causes a decrease in both humoral and cell-mediated immunity. CD4^+^ cells release cytokines that contribute to the activation of antigen-presenting, phagocytic, natural killer, and cytotoxic T cells against pathogenic infections. They also support the conversion of B lymphocytes into long-lived plasma and memory B cells. Ultimately, the decline in CD4 + levels predisposes patients with AIDS to a higher risk of opportunistic infections due to pathogens [38–41]. Therefore, the differences between patients with AIDS and the control group were reasonable, indicating that mNGS detection may be more helpful than traditional methods in detecting a wide range of AIDS-related pathogens or even multiple infections. Furthermore, it was shown that mNGS carried a higher diagnostic positivity rate than bacterial (*P* value < 0.0001, Chi-square test) and fungal (*P* value < 0.0001, Chi-square test) culturing methods. Because mNGS was able to detect pathogens from patients’ tissue displaying potential infection and colonization, while culture results remained negative. Coincidentally, Wang et al. reported that mNGS-based detection of mixed pulmonary infections increased the sensitivity rate by approximately 83.3% compared with that of culturing (97.2% vs. 13.9%; *P* < 0.01) [42].

Theoretically, the high sensitivity of mNGS, which is strongly affected by the sequencing depth, could overcome the dilemma of low levels of microbial sequences. Therefore, more potential pathogens were detected and classified, which made an excellent contribution to the diagnosis of multiple infections and development of appropriate treatment strategies. An increasing number of case reports have revealed that mNGS has been successfully used as a diagnostic tool for infectious diseases caused by *Streptococcus suis* [43], *P. jirovecii* [44, 45], *cytomegalovirus* [46], and *T. whipplei* [45] in patients with AIDS.

However, mNGS may not be able to identify some specific culturable pathogens. In our study, mNGS missed *Mycobacterium* (four cases) and *Aspergillus spp.* (one case) and performed poorly in the detection of *Mycoplasma spp.* (two cases), *U. urealyticum* (two cases), and *M. kansasii* (one case) (Table 1). Among the six *P. jirovecii*-positive samples, five were not reported using mNGS. This is possibly due to the relatively low abundance of these pathogens within the samples or hindrance of the commensal microbiome in the respiratory tract. Because of the use of PCR-enriched sequences in library construction (Fig. 1), some sequences with less content may fail to be amplified, resulting in the loss of some information and missed detection of pathogens. The introduction of third-generation sequencing platforms such as Pacific BioSciences (PacBio) and Oxford Nanopore Technology (ONT) may provide the full structure of the microbial genome and reduce the sequencing bias caused by PCR amplification [47]. However, the high cost of third-generation sequencing has restricted its widespread application in clinical diagnosis.

Another limitation of mNGS is that while it identifies, it cannot discriminate the pathogenicity status. It could be confusing and even misleading for doctors reporting diagnoses and deciding clinical therapeutic strategies. Herein, 16 cases of AIDS patients carried a specie of confirmed pathogen with pulmonary pathogenicity diagnosed by mNGS and re-identified by the gold standard method. In addition, it lacks a standard criterion for explaining and reporting the number of reads, coverage of bases, sequencing depth, mNGS-positive or -negative results, the concentration of organisms, and functional differences, which strongly affect the clinical application of mNGS-based diagnosis [48]. Respiratory colonizing microorganisms (e.g. *Staphylococcus aureus*, *e. aeruginosa*, *A. baumannii*, *Herpes simplex virus type 1*, *Human gammaherpesvirus 4* or *Human cytomegalovirus*) existed in equilibrium with the host by evading clearance of the immune system [49]. However, people acquired immunodeficiencies increased susceptibility to both invasive disease and higher density of colonizers, which were further transformed into pathogens and induced pulmonary disease development [50]. We must clarify that these 6 species in AIDS patients should be considered as potential pathogens and given priority attention even in cases with relatively low level of unique reads detected by mNGS. In contrast, cases with less than 50 unique reads in non-AIDS patients (without gold standard validation or clinical characterization) might be colonizers. Actually, *oral streptococci*, *e*, *salivary streptococci*, *Prevotella spp.*, *e* and *Rhodobacter spp.* were common colonizing microorganisms of the human oral cavity, and confirmation of the causative agents of pulmonary infections was subject to exclusion of contamination during sampling and adequate clinical diagnosis [51]. Thus, the clinical application of mNGS does not mean that doctors do not need a conventional forensic pathological diagnosis in forensic medicine; but it could improve diagnostic efficiency and serve as a supplementary method to culture. However, the explanation and reporting of mNGS results can be confusing and unsubstantiated.

Furthermore, the relatively small sample size in the present study may have affected the accuracy of the mNGS assessment. For example, only 75 patients with AIDS from the same area participated in this investigation, and one microbiome sample was tested per individual. Generally, pathogenic diversity and composition fluctuate according to sex, area, time, and other factors. In addition, some of the detected pathogens were characterized by low-level reads of mNGS data. A set of probable false-positive results that stem from experimental errors should be avoided by future PCR or digital PCR validations. Moreover, analyses of serology, drug resistance, phylogenetic evolution, and other biological characteristics are lacking. This limits the extent to which we can statistically identify actual pathogens based on the differences in different detection technologies and the power of the correlational analyses between microbiota and functional differences.

## Conclusion

In conclusion, mNGS has emerged as a promising tool for collecting the sequence information of various pathogenic microorganisms in an unbiased manner. Subsequent bioinformatics’ analyses of the microbiome can provide a reliable basis for accurate diagnosis, real-time monitoring, and effective therapy. The results of our study strongly support carried a less turnaround time and higher diagnostic positivity rate in the routine clinical diagnosis of patients with AIDS and unknown pulmonary pathogenic infection, especially when the results of conventional laboratory-based diagnostic screening are negative or the patients have multi-pathogenic infections. To achieve early diagnosis, standard operating procedures and management decisions must be established according to well-designed trials to eliminate or reduce biases between samples.

## Data Availability

The datasets used and/or analysed during the current study are available in the NCBI Bioproject repository, [PRJNA891782].

## Abbreviations

AIDS: Acquired immunodeficiency syndrome
mNGS: metagenomic next-generation sequencing
HIV: Human immunodeficiency virus
PCR: Polymerase Chain Reaction
PacBio: Pacific BioSciences
ONT: Oxford Nanopore Technology
